# Protein comparability assessments and potential applicability of high throughput biophysical methods and data visualization tools to compare physical stability profiles

**DOI:** 10.3389/fphar.2014.00039

**Published:** 2014-03-12

**Authors:** Mohammad A. Alsenaidy, Nishant K. Jain, Jae H. Kim, C. Russell Middaugh, David B. Volkin

**Affiliations:** Department of Pharmaceutical Chemistry, Macromolecule and Vaccine Stabilization Center, University of KansasLawrence, KS, USA

**Keywords:** comparability, high throughput, biophysical, protein, stability, monoclonal antibodies, formulation

## Abstract

In this review, some of the challenges and opportunities encountered during protein comparability assessments are summarized with an emphasis on developing new analytical approaches to better monitor higher-order protein structures. Several case studies are presented using high throughput biophysical methods to collect protein physical stability data as function of temperature, agitation, ionic strength and/or solution pH. These large data sets were then used to construct empirical phase diagrams (EPDs), radar charts, and comparative signature diagrams (CSDs) for data visualization and structural comparisons between the different proteins. Protein samples with different sizes, post-translational modifications, and inherent stability are presented: acidic fibroblast growth factor (FGF-1) mutants, different glycoforms of an IgG1 mAb prepared by deglycosylation, as well as comparisons of different formulations of an IgG1 mAb and granulocyte colony stimulating factor (GCSF). Using this approach, differences in structural integrity and conformational stability profiles were detected under stress conditions that could not be resolved by using the same techniques under ambient conditions (i.e., no stress). Thus, an evaluation of conformational stability differences may serve as an effective surrogate to monitor differences in higher-order structure between protein samples. These case studies are discussed in the context of potential utility in protein comparability studies.

## Introduction to comparability assessments

Protein based drugs are structurally complex, highly specific macromolecules used therapeutically to compensate for body deficiencies (e.g., hormones and clotting factors), as medical treatments (e.g., cytokines and monoclonal antibodies) as well as to prevent diseases (e.g., polyclonal antiserum and certain vaccines) (Aggarwal, 2012). A protein molecule's structural complexity makes it quite challenging to preserve biological activity and stability throughout manufacturing, storage and administration. Protein drug molecules are prone to different degradation mechanisms (chemical or physical) during manufacturing and storage, which ultimately can lead to the loss of potency (through structural alterations) as well as potentially increases in immunogenicity reactions upon administration (Rosenberg, 2006; Murphy and Tsai, 2006; Weert and Møller, 2008; Manning et al., 2010). Chemical degradation pathways involve covalent bond modifications, as in Asn deamidation, Asp isomerization, Met oxidation, polypeptide chain proteolysis and disulfide bond shuffling (Manning et al., 2010). Physical degradation, on the other hand, includes distinct pathways such as protein structural alterations (Murphy, 1995), surface adsorption (Bee et al., 2011), aggregation (Narhi et al., 2012) and precipitation (Wang, 2005). There are many causes leading to the physical and chemical degradation of proteins including exposure to different environmental stresses [e.g., agitation(Brych et al., 2010), UV light (Davies, 2003) and temperature (Banks et al., 2012)] as well as changes in solution and formulation conditions [e.g., pH (Hari et al., 2010), ionic strength (Majhi et al., 2006) and additives (Hamada et al., 2009)]. A better understanding of the possible degradation mechanism of a protein therapeutic is a key step in the formulation development of safe and effective product candidates to be evaluated in clinical trials.

Currently there are more than 150 protein-based drugs approved by the FDA (1996) and EMA including ~35 monoclonal antibodies (mAbs). In addition, more than 900 medicines and vaccines are under clinical development, with 30 mAb candidates in late-stage trials (Aggarwal, 2012; Reichert, 2013). There has been a concomitant increase in demand for more production capacity to make larger amounts of protein drugs, challenging manufacturers to develop new technologies and to scale-up their manufacturing processes. The biopharmaceutical industry and government regulators need an efficient and scientifically based approach to evaluate the effect of process changes and production scale-up on the structural integrity, physicochemical identity and clinical safety and efficacy of protein drug candidates. This first led to the development of “well characterized biotechnology products” and “comparability” regulatory guidance documents in 1996. These general concepts of comparability were then further defined and expanded by additional guidances from the European Medicines Agency (EMA) in 2003 (Committee for Proprietary Medicinal Products), the International Conference on Harmonization (ICH) (Q5E) in 2003 (ICH Q5E, 2005) and by the World Health Organization (WHO) in 2009 (Expert Committee on Biological Standardization, 2009).

The primary and higher order structures of protein drug molecules are delicate in nature, especially compared to small molecule drugs, and are highly sensitive to environmental changes and stresses in general. Changes in the manufacturing process for a protein therapeutic may occur during clinical development or after commercialization. These changes frequently occur in either the manufacturing steps of the active drug substance (e.g., facility, equipment or process changes) or in the finished drug product (e.g., formulation excipients, container-closure system, dosage form changes) (Chirino and Mire-Sluis, 2004; Federici et al., 2013). The manufacturer may apply changes in their manufacturing processes for variety of reasons such as improving yields and purity, ensuring better patient convenience, or facilitating compliance with new regulations. Comparability studies are performed to assess the effect of these process and product changes on the “critical quality attributes” (CQAs) by comparing the pre and post-change protein products through a series of tests performed in a head-to-head fashion. Critical quality attributes are defined as the collective product qualities defining the identity, purity, potency, safety and stability of the protein drug product (ICH Q5E, 2005).

**Figure 1:**
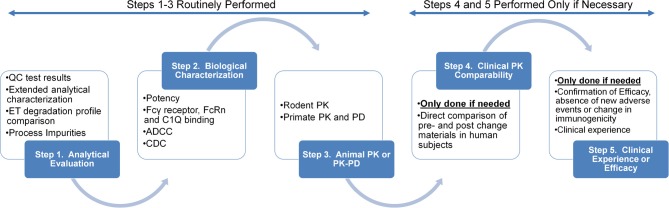
**Summary of step-wise nature of studies performed in a typical comparability assessment of a protein based drug candidate**. Reproduced from Federici et al. (2013) with permission from Elsevier.

The assessment of comparability between the pre- and post-change product relies on the ability to show experimentally the samples are “highly similar” in terms of physiochemical and biological characteristics, degradation profile, pharmacokinetics and immunogenicity (ICH Q5E, 2005). Physiochemical characterizations, biological assays and stability degradation profiles of the drug product are considered the cornerstone of a comparability exercise (Figure 1). The ability to establish highly similar analytical profiles determines the need for additional animal or clinical evaluations such as pharmacokinetic, efficacy and/or immunogenicity studies (ICH Q5E, 2005).

### Physiochemical characterization

Extensive physiochemical analytical characterization of the pre- vs. post-change active pharmaceutical ingredient (i.e., protein itself) and the final drug product (final dosage form including protein, excipients, and primary container) is typically the first step of a comparability evaluation. Peptide mapping is typically the initial step, usually used to assess the integrity of the amino acid residues and sequence within a specific protein molecule. This technique involves the chemical or enzymatic treatment of the protein to generate a specific set of peptide fragments, which in turn are separated using reversed phase high performance liquid chromatography (RP-HPLC) equipped with diode-array ultraviolet (UV) for detection. Isolated peptide fragments are also mass characterized using online mass spectrometry. Peptide mapping has proven to be an especially valuable technique in comparability studies, since each protein upon digestion with specific proteolytic enzymes will give specific peptide fragments that are used as a high resolution fingerprint for the protein under evaluation (Skrlin et al., 2010; Lubiniecki et al., 2011; Berkowitz et al., 2012; Li et al., 2013). Comparing the peptide map profiles for the pre and post-change proteins can show alterations in the protein polypeptide backbone such as fragmentation or changes in individual amino acid residues (e.g., mutation, oxidation, deamidation, or post-translational modifications). The correct disulfide pairing can also be evaluated using the same technique by comparing peptides under reduced and non-reduced conditions.

Various post-translational modifications (PTMs) such as N-glycosylation, O-glycosylation and/or C-terminal lysine clipping, are frequently observed upon changing the cell-culture manufacturing processes. This is partially responsible for the heterogeneous nature of pharmaceutical proteins, since they are usually produced by cells in culture (Walsh and Jefferis, 2006). Additionally, for glycoproteins, the nature of glycans and the extent of glycosylation (e.g., erythropoietin is 40% N-glycosylated vs. 2% N-glycosylation in an IgG1 monoclonal antibody) (Beck et al., 2012) may be affected by process changes. Mass spectrometry based techniques are again frequently used to obtain insight into a glycoprotein's glycosylation pattern. Mass spectrometric ionization based techniques such as matrix-assisted laser desorption/ionization (MALDI-MS) and electrospray ionization (ESI-MS) are frequently used for this purpose (Zaia, 2008; Brooks, 2009; Beck et al., 2012; Pompach et al., 2012; Desaire, 2013). A more detailed picture of the glycan structure and composition can be achieved using oligosaccharide mapping in which the glycan residues are enzymatically cleaved from the protein and then labeled with a fluorescent tag (e.g., 2-aminobenzamide is frequently used) which is used for detection (Leymarie and Zaia, 2012; Baković et al., 2013).

Alterations in glycosylation patterns of proteins as a result of manufacturing changes have been reported (Lubiniecki et al., 2011). The role of glycosylation differences on the stability of erythropoietin have also been investigated (Narhi et al., 1991). Glycosylated erythropoietin was found to be more resistant to denaturant and acidic pH induced unfolding compared to the non-glycosylated form of the protein. Three intact IgG1 mAbs were compared before and after glycan removal using a variety of analytical techniques (Zheng et al., 2011). An increase in fluorescence signal upon deglycosylation was observed indicating structural perturbations, a conclusion that was further reinforced by DSC results indicating a structural change in the C_H_2 region of the IgG1 mAbs. A similar study looked at the influence of a series of differentially glycosylated IgG1-Fc mAbs with varying glycan length generated through an enzymatic reaction on the conformational stability (Mimura et al., 2000). Using DSC, glycosylation length was found to play a role in IgG1 conformational stability specifically in the C_H_2 domain where the N-linked glycosylation site resides. In addition to influencing conformation, glycosylation was found to have an effect on the protein's susceptibility to proteolysis. The existence of the glycan and the type of terminal sugar in the glycan residue were reported to influence the susceptibility of an IgG to papain digestion (Raju and Scallon, 2006, 2007).

Ensuring native-like higher order structure (secondary, tertiary and quaternary structures) in a protein produced from both pre- and post-change manufacturing processes is essential since the overall three dimensional configuration not only defines a protein's stability and functionality, but also its efficacy and safety when being used as a therapeutic. Differences in higher order structures could stem from physical changes in structural integrity (i.e., partial unfolding, conformation changes and aggregation) as well as from modifications in either the side chains (mutation, deamidation) and/or PTMs such as glycosylation. These differences in higher order structure (HOS) are sometimes only detected during long-term storage or accelerated storage which may involve changes to the surrounding environment (e.g., temperature shifts or agitation). In addition to the properties of the protein molecule itself, pharmaceutical additives (i.e., excipients such as salts, surfactants, sugars, etc.), solution conditions (such as pH and ionic strength) as well as the nature of the final dosage form (liquid vs. lyophilized) can also impact the HOS of a protein.

The assessment of higher order structural integrity and conformational stability of proteins in comparability studies is best performed using multiple biophysical and calorimetric techniques. Far-ultraviolet circular dichroism (CD) and Fourier transform infrared spectroscopy (FTIR) have been widely used to analyze the overall secondary structure content of proteins. These methods produce distinctive signals that correspond to the different secondary structural folds (i.e., α-helices and β-sheets). Detailed studies regarding the use of CD spectra for structural comparisons in comparability studies have been published recently (Li et al., 2011; Teska et al., 2013). Common techniques for the evaluation of the overall tertiary structure of proteins are near UV absorbance measurements, fluorescence spectroscopy and near-UV CD. Owing to the unique fluorescence and absorbance properties of aromatic amino acid residues (tryptophan, tyrosine and phenylalanine) and their sensitivity to the polarity of their local environment, changes in a protein's structural integrity and conformational stability upon partial or full unfolding can be monitored. Furthermore, extrinsic fluorescence dyes (e.g., 1-anilino-8-naphthalenesulfonate, Sypro orange, etc.) have been employed to monitor partially unfolded intermediates (e.g., molten globular states) which have been often shown to play a role in initiating protein aggregation events (Hari et al., 2010; He et al., 2012; Sahin et al., 2012). Such dyes have the valuable property of being relatively non-fluorescent in polar environments (such as water) but become highly fluorescent in less polar, more hydrophobic milieu such as protein interiors. These features allow scientists to examine the onset of structural unfolding of a protein as more apolar regions within a protein are exposed when a protein is stressed (e.g., during heating or agitation). Differential scanning calorimetry (DSC), another powerful analytical technique that can be used to monitor protein conformational stability, measures heat capacity differences between the protein solution and a reference buffer as a function of temperature. As a protein's structure begins to alter, heat capacity increases resulting in an endothermic peak centered at the thermal melting temperature (Tm, the midpoint of a protein unfolding event). As part of a comparability study, DSC can also provide information about the overall conformation stability of the proteins under evaluation. Structural alterations in a protein as a result of manufacturing process changes can be detected in the form of a Tm shift or a change in the shape of the endothermic peak. Multi-domain containing proteins (such as a monoclonal antibody) show multiple endothermic peaks in a given DSC thermogram. Structural changes in one of these domains can be reflected in corresponding changes to the associated DSC peak and further studied by deconvolution.

A limited number of case studies evaluating the effect of changes in manufacturing processes on protein structure and function from an analytical comparability perspective have been published. In one study, the effects of changing the final dosage form from a lyophilized formulation to a liquid dosage form, in addition to the impact of transferring the bulk drug substance to the commercial site, were evaluated for two monoclonal antibodies (Lubiniecki et al., 2011). An increase in deamidation at a specific Asn site for one of these mAbs was observed which did not notably effect the structural integrity, biological potency or the pharmacokinetic profile of the antibody in animal models. This Asn deamidation was attributed to the transfer of the bulk process to the commercial site at an increased scale which required longer hold times in solution. Additional analytical evaluations of the mAb's conformational stability as well as functionality using multiple analytical methods were performed, with no differences observed between the two dosage forms of both mAbs. The impact of changing formulation composition on protein conformational stability has been addressed in a comparability study (Cauchy and Hefford, 2010). In this work, the effect of formulation exchange on a human growth hormone was evaluated. The authors concluded that the formulation change affected the structural integrity of the protein and additional considerations needed to be given regarding the choice of final excipients. Selecting an appropriate formulation for insulin was emphasized in another study as they reported that changes observed on the physical and chemical stability of insulin (due to changes in its crystallization process) were dependent on the raw materials and excipients used in formulation (DeFelippis and Larimore, 2006).

### Biological activity

Techniques used to characterize the biological activity of a protein serve as a critical complement to physiochemical assays in confirming the correct higher order structure of a protein. Determining bioactivity for monoclonal antibody based protein drugs is usually done using a variety of *in vitro* binding assays such as enzyme-linked immunosorbent assays (ELISA) and surface plasmon resonance (SPR). Another group of bioassays, that directly measures biological functionality, are often also used with protein drugs including *in vitro* cell proliferation and *in vivo* animal models. Using functionality bioassays (if feasible) in comparability studies is typically preferred over simple antigen binding assays because some degraded, modified protein molecules could still bind non-specifically to the target molecule in a simple binding assay. Protein molecules containing multiple regions with different functionalities should have multiple functionality and/or binding bioassays reflecting each and every structural region. For example, monoclonal antibodies are composed of two antigen binding sites (Fab), responsible for antigen binding, and one crystallizable region (Fc), responsible for immune effector functions. Each of these regions may need to be tested for its ability to bind to its specified target to perform its desired function.

Case studies examining protein functional activity changes due to changes in manufacturing process have been reported. An oxidized form of the protein filgratism (a human granulocyte-colony stimulating factor used primarily for patients with severe neutropenia) was found to lose 75% of its potency, whereas, filgratism dimer had 67% potency compared to the intact molecule (Cauchy and Hefford, 2010). In addition to influencing protein conformation, glycosylation can play a critical role in defining biological functionality. In the case of a mAb, the presence of a core fucose unit in the N-linked oligosaccharide was found to be responsible for decreasing the protein's antibody-dependent cellular cytotoxicity (ADCC). This highlights the importance of glycan identification in comparability studies for pre- and post-change mAb products (Shields et al., 2002). In another example, TNK-Tissue Plasminogen Activator (TNK-tPA) is a glycosylated protein which contains four glycosylation sites, three of which are N-linked and the other O-linked. In a comparability study (Jiang et al., 2010) of a biosimilar molecule under development, the biosimilar protein had a similar glycosylation pattern to the innovator drug product, except for one N-glycosylation site which was found be only partially glycosylated. The close proximity of the partially glycosylated glycan to the active site raised concerns about its influence on the clot lysis biological activity, resulting in the need for more detailed studies to investigate such effects. Another marketed protein product called Myozyme (Alglucosidase alpha), used to treat patients with Pompe disease, that was produced at two different sites by the same company was found to be dissimilar in terms of biological activity (Kozlowski et al., 2011). These differences in biological activity were attributed to glycosylation differences between proteins from the two manufacturing sites.

### Accelerated and forced degradation stability studies

Forced degradation studies comprise a group of analytical tests applied to the drug product to elucidate the physicochemical mechanism(s) of protein degradation. Accelerated stability studies measure the rate of given degradation process over time at various temperatures in specific formulations and primary containers. Evaluating the effect of storage period, excipients and environmental stress on the accelerated (and long term) stability of protein drug products is an essential part of formulation development and comparability evaluations. Protein drugs encounter different environmental stresses during production, storage, shipment and patient administration. Thus, accelerated stability studies and forced degradation studies are needed to determine the stability profile of a protein drug by subjecting the protein to various stresses including elevated or changing temperatures, freezing, thawing, agitation, oxidative environment, light, and the presence of different interfaces and pH changes. (Britt et al., 2012; Cordes et al., 2012; Miller et al., 2013; Wang et al., 2013a) The design of successful forced degradation and accelerated stability studies depends on having the appropriate analytical tools to detect, quantify, and characterize any degradants, impurities and side products generated during these studies.

Regulatory guidelines for stability studies that may be applied to comparability assessments can be found in ICH Q3C (2003), ICH Q5C (ICH Q3C, 1996) and ICH Q5E (2005). For example, the ICH Q3C guideline mentions the importance of developing analytical assays to quantify residual impurities of xylene leaching from rubber stoppers during a freeze-drying process. Similarly, the ICH Q5E guideline talks about divalent cations leaching from vial stoppers which in turn activate trace amounts of proteases leading to the degradation of a protein drug. Designing a successful comparability stability study also depends on the type of process or product change being evaluated. For instance, a change in the final dosage form from a liquid to a lyophilized drug product will generally result in a more stable product and thus would not necessarily be expected to have a similar stability profile. In this case, showing similarity of degradant type would be of primary interest. In an opposite scenario, changing from a lyophilized dosage form to a liquid one, would not only require evaluating the types of degradants but also testing the protein drug product under additional stress conditions (e.g., mechanical stress) to get additional insight into the aggregation behavior of the liquid formulated protein drug.

Exposure to new surfaces from changing the primary containers or formulation additives has been reported to induce protein aggregation and oxidation in some protein drug products. In one study (Lubiniecki et al., 2011), an assessment of the impact of changing the primary packaging from a vial to a pre-filled syringe in terms of conformational integrity and stability of two mAbs was evaluated. Similar molecular structure, biological activity and degradation profiles were seen for both mAbs in a liquid formulation filled in the vials and syringes with the one exception: a small but statistically significant difference in subvisible particle levels was noted and attributed to the presence of trace amounts of silicon oil in the syringes. This has been reported to have an undesirable effect on protein stability and aggregation (Jones et al., 2005; Thirumangalathu et al., 2009). As a final example, the biophysical stability of three lots of an IgG1 mAb that were manufactured and filled at different locations was assessed (Maity et al., 2012). These three lots were incubated for multiple time periods (0, 8, and 16 weeks) under either refrigeration (4°C) or heat stressed conditions (40°C). No differences between the different lots of protein in terms of conformational stability as well as in aggregation behavior were detected for the refrigerated lots. In contrast, variations between the different lots stored at 40°C at different time points were seen using extrinsic (using ANS as a dye) and intrinsic (Trp) fluorescence spectroscopy. Different levels of aggregation were noted by static light scattering (SLS) and size exclusion high performance liquid chromatography (SEC).

### Pharmacokinetic studies

In addition to the analytical comparability testing described above, pharmacokinetic (pK) studies may need to be evaluated, in the form of either non-clinical or clinical studies, in which the pharmacokinetic profile of the protein drug is evaluated using animal models (rodents or primates) or in humans, respectively. In cases involving an observed non-similarity between the products under comparison, it may be necessary to perform pharmacokinetic studies in human subjects to demonstrate comparability (Putnam et al., 2010). These pharmacokinetic studies are performed using the same route of administration and dose regimens providing comparative results (e.g., absorption, bioavailability as well as elimination parameters like clearance and elimination half-life) that should be highly similar for the two samples being compared (Putnam et al., 2010). Changes to a protein's glycosylation pattern, charge distribution and aggregation profiles upon manufacturing changes could have a measurable effect on its pharmacokinetic profile, which may or may not have an effect on the drug pharmacodynamics, efficacy and safety profiles. The evaluation of such pK affects are almost always carried out first using animal models (e.g., either rodents or primates), and if results are inconclusive, human clinical pharmacokinetic comparability studies may be necessary.

Glycosylation can play a central role in defining the circulation and half-life in the human body for glycoproteins. In one example, using a rat animal model, 50% loss in the amount of a circulating protein (Ceruloplasmin, a copper carrying protein in the blood) was observed after a 20% reduction in sialic acid content of the glycan attached to the protein (van den Hamer et al., 1970). In some glycoproteins lacking sialic acid, it has been shown that they have a higher affinity for mannose receptors in liver cells, leading to a faster clearance and catabolism compared to the fully sialylated ones (Stockert, 1995). The same fate was observed for antibodies containing mannose terminated N-glycans (Kanda et al., 2007; Goetze et al., 2011). Multiple comparability studies have examined glycoproteins with different distributions of glycosylation patterns due to a process change (especially cell-line and cell culture changes). In one study, the amount of sialic acid, in addition to the relative distribution of neutral oligosaccharides, were different between the pre and post-change for mAb proteins after changing the producing cells form a murine myeloma cell line (NS0) to Chinese hamster ovary cells (CHO) (Kilgore et al., 2008). Another study demonstrated that using a different clone of the same cell line, or changing the cell culture components, resulted in a significant increase in acidic glycoforms of a mAb compared to the original cells (Lubiniecki et al., 2011).

Pharmacokinetic parameters have also been reported to be sensitive to changes in the overall charge and aggregation state of a protein drug. Proteins with specific charge heterogeneity profiles have been shown, after modifying the manufacturing method, to have a different charge distribution profile (He et al., 2009). Changes in the overall charge state of a protein could alter the electrostatic and hydrophobic interactions between the protein and negatively charged cell membranes leading to a different pharmacokinetic profile (Khawli et al., 2002). Using animal studies, minor shifts in the p*I* of a protein (in the range of 0.1-0.2 p*I* units) have been demonstrated to have no effect on the pharmacokinetic profile (Harris, 2005). In contrast, shifts of above 1.0 p*I* units may have a measurable effect. In one study, protein cationization resulted in longer tissue retention times and a faster rate of clearance (Hong et al., 1999). Protein anionization tended to cause a decrease in tissue retention time but still increased whole body clearance (Dellian et al., 2000) (demonstrated in rats and mice, respectively). At the molecular level, changes in the charge heterogeneity profile of a protein molecule could arise from alterations in the primary sequence of the protein as a result of amino acid mutations or chemical degradation (e.g., Asn deamidation) (Sosic et al., 2008). Post-translational modifications (e.g., sialic acid present on the glycan residues and C-terminal lysine) can also contribute to the charge heterogeneity profile of a protein (Sosic et al., 2008). Protein aggregation may affect pharmacokinetics profiles both directly or indirectly through the induction of neutralizing antibodies. For example, insulin oligomers have been reported to have a slower absorption and bioavailability upon subcutaneous administration compared to non-aggregated form of the protein (Pezron et al., 2002).

### Clinical immunogenicity studies

Changes to the native structure of a protein could potentially reveal or create new antigenic sites that could stimulate an immune response. An unwanted immune response against a protein drug can be stimulated by many factors that are generally divided into two categories: extrinsic and intrinsic. Patient immunogenic susceptibility and patient health status (administering co-medications) in addition to the dose and route of administration are all considered extrinsic factors that may be related to the immunogenicity of a protein based drug (Bal et al., 2012). Intrinsic factors are those related to the protein or the drug product itself including aggregation propensity, impurities (e.g., residual host cell protein not removed by the purification steps) and leachables (from container-closure systems, which may or may not be immunogenic by themselves but could induce protein aggregation) (Ohkuri et al., 2010; Johnson and Jiskoot, 2012; Rosenberg et al., 2012). The consequences of an immune reaction against a protein drug could vary significantly, ranging from clinically minor to severe, life-threatening effects. In one unfortunate case, a specific erythropoietin-α drug product formulation (Eprex®) was associated (in 1998 and peaked in occurrence in 2002) with pure red cell aplasia (PRCA) (Gershon et al., 2002). An increase in neutralizing antibody titers in patients with chronic renal failure against the protein drug was found, which was correlated with changes made to the product formulation (Boven et al., 2005). Human serum albumin (HSA), used as a stabilizer, was removed from the original formulation and substituted by polysorbate 80 surfactant. The resulting instability has been rationalized in one report by the leaching of compounds (Seidl et al., 2012) from the rubber plunger of the pre-filled syringe causing the protein to unfold and aggregate. Another theory suggested that polysorbate 80 formed micelles with the protein resulting in an adjuvant-like nanoparticle that stimulated the immune system (Schellekens, 2003). The route of administration can also play a critical role in product immunogenicity. It was found that patients who received Eprex® through an intravenous injection (IV) did not get PRCA unlike those that received the drug subcutaneously (SC) (Schellekens, 2008).

Glycoproteins bearing different kinds of monosaccharides or different linkages than the naturally occurring ones could potentially stimulate the immune response as well. These glycan differences are introduced by various mammalian cell lines used to produce recombinant proteins. For example, the presence of galactose-α-1, 3 glycans has been associated with anaphylactic shock and an immune response in patients using cetuximab and bovine thrombin, respectively (Schoenecker et al., 2000; Chung et al., 2008). N-glycolylneuraminic acid (Neu5Gc or NGNA) is a sialic acid glycan that contains an additional oxygen atom compared to the naturally occurring N-acetylneuraminic acid (Neu5AC or NANA) found in humans. The presence of this sialic acid form (NGNA) has been associated with immunogenicity risks (Hokke et al., 1990). A comparison between cetuximab and panitumumab (both antibodies specific for binding epidermal growth factor receptor), concerning the addition of Neu5Gc to the glycan structure during expression in cell culture, showed that cetuximab was only incorporating NGNA into the glycan structure (Ghaderi et al., 2010). The addition of NANA to the culture media helped reduce the incorporation of NGNA to the glycoprotein drug. Finally, complete deglycosylation of a glycoprotein has been associated with an increasing incidence of immunogenicity. Mechanisms underlying such an effect are not fully understood, however, aggregation resulting from exposure of normally hidden hydrophobic patches, decreasing solubility and exposure of new antigenic sites (or a combination of factors) has been proposed. For example, immunogenicity due to deglycosylation was reported for interferon-β (Rudick et al., 1998; Runkel et al., 1998)and granulocyte macrophage colony-stimulating factor (GM-CSF) (Gribben et al., 1990).

Protein aggregation is thought to have a significant impact on the immunogenicity potential of protein drugs. Protein aggregation can be exacerbated by multiple factors including changes in solution conditions (e.g., pH, salts, excipients, etc.) (Majumdar et al., 2013; Manikwar et al., 2013; Wang et al., 2013a) and exposure to various environmental stresses (e.g., temperature, agitation, freeze-thaw, etc.) (Thirumangalathu et al., 2009; Cordes et al., 2012; Sahin et al., 2012). An immunogenic response in patients receiving an interferon alpha was attributed to the presence of aggregated species in the drug product, and was seen after alterations in the formulation components (Braun et al., 1997). A positive correlation between protein aggregation and immunogenicity has been shown as well for other therapeutic proteins, such as insulin (Robbins et al., 1987), human growth hormone (Moore and Leppert, 1980) and bovine serum albumin (Cleland et al., 1993) confirming the important role of protein aggregate detection and characterization in protein drug products. (Wuchner et al., 2010; Jiskoot et al., 2012)

Immunogenicity prediction in humans using either *in vitro* models or animal studies is difficult, and the development of new approaches to obtain more reliable immunogenicity tests remains highly desirable (van Mierlo et al., 2013). The need to evaluate proteins after manufacturing changes for immunogenicity becomes even more important for products with a past history of immunogenicity (the pre-change product), as well for those protein products with manufacturing changes that are considered extensive (i.e., alteration of the cell line) or those that result in observations of increased levels of aggregates or particles.

### Current analytical challenges in comparing protein higher-order structure

The type and extent of any comparability exercise depends on the nature of the protein (size, complexity, and microheterogeneity), the magnitude and type of manufacturing changes, and the analytical tools available to monitor the structural integrity of a protein. An in-depth understanding of the protein including its inherent susceptibility to the different chemical and physical degradation mechanisms, combined with accumulated clinical and manufacturing experience with a product, is essential for a successful comparability study. Accurate structural determination for protein-based drugs is a difficult task for the pharmaceutical and biotechnological industry, in contrast to small molecule drugs, because of their complex three-dimensional structures, sensitivity to their environment and their inherent microheterogeneity. Major advances have been made in developing analytical tools for primary structure analysis, ranging from chromatographic (size exclusion, reversed phase and ion-exchange HPLC) and electrophoretic (capillary isoelectric focusing and capillary sodium dodecyl sulfate) separation methods that are typically linked with mass spectrometry detection (intact molecular weight, peptide maps, and oligosaccharides maps) to accurately characterize a protein's primary structure including any post-translational modifications.

Physiochemical characterization of the higher-order structural integrity of a protein when two slightly different but homologous drug products are being compared is one of the essential elements of a comparability study. In fact, the current regulatory expectation for performing functional biological potency assays to ensure biological activity is considered a necessary check on the higher order structural integrity of a protein. Although various lower resolution analytical techniques have been used and developed to test higher order structural comparability between two protein drugs (e.g., CD, fluorescence, and DSC as discussed above), the use of higher resolution analytical techniques such as NMR, X-ray crystallography, cryo EM and H/D exchange mass spectrometry that provide structural details about a protein's folded structure and its dynamic behavior remains limited as part of analytical comparability studies. Although these techniques are very powerful in terms of obtaining detailed information about a folded protein's structure, their use has been restricted due to some drawbacks including protein size and formulation composition (presence of excipients) in addition to being technically challenging, expensive and time consuming techniques. Research is currently being performed to overcome these drawbacks. For example, the effect of different anions and excipients on an IgG1 mAb local flexibility was evaluated using H/DX-MS (Majumdar et al., 2013; Manikwar et al., 2013). An increase in the local flexibility of a certain peptide fragment in the C_H_2 domain of the mAb was seen upon using thiocyanate (as an anion) and arginine (as an excipient) that was correlated with decreased thermal stability of the C_H_2 domain and an increase in aggregation propensity. These results demonstrate the intricate interrelationships between different excipients and their effect on protein dynamics and physiochemical stability of a monoclonal antibody. Recent advances in both H/DX-MS (Houde et al., 2011) and NMR (Amezcua and Szabo, 2013) have shown promising potential in comparability studies, but have not yet reached the level of routine use.

More well established, commonly available, lower resolution biophysical techniques such as CD, intrinsic and extrinsic fluorescence spectroscopy, DSC, static light scattering and turbidity measurements are widely used for the evaluation of secondary/tertiary structure and colloidal stability of a protein within a pharmaceutical dosage form containing formulation components. These biophysical methods can be setup as high throughput assays in which analysis can be performed automatically across various environmental conditions such as changes in solution pH and temperature. Elevated temperatures and extreme pH conditions are frequently used as stress factors to evaluate the overall conformational sensitivity of proteins during formulation development, but the use of these types of data sets for comparability analysis has not yet been extensively explored. By using data sets acquired from multiple low-resolution biophysical techniques that monitor different aspects of a protein's higher-order structural stability as a function of environmental stress, differences in structural integrity may potentially be detected when these differences are not readily apparent when monitored using lower resolution methods under non-stressed conditions (i.e., at low temperatures under neutral pH conditions). Additionally, the evaluation of conformational stability differences may not only be an effective surrogate to monitor differences in higher-order structure between protein samples, but also a useful complement to traditional accelerated stability and forced degradation studies often used in analytical comparability studies as described above.

## High throughput biophysical methods and data visualization techniques

Although lower resolution biophysical techniques such as CD, intrinsic/extrinsic fluorescence spectroscopy, DSC and static light scattering measurements are commonly used to monitor the structural stability of protein-based drugs, no single technique provides sufficient information to establish the higher order structural integrity of complex macromolecules such as proteins. Therefore, the use of more than one technique is generally needed for better characterization. The multi-dimensional nature of such analysis makes collection, analysis and visualization of larger data sets desirable. Historically, data analysis of protein conformational stability data was performed by either visual inspection or data fitting of thermal unfolding curves to sigmoidal functions of results from individual instruments. These types of approaches can result in data interpretation that is not only subjective, but limited in terms of their scope and utility.

In 2003, a new data visualization method was introduced as a tool for analysis of protein physical stability data obtained from high throughput biophysical measurements (Kueltzo et al., 2003). This method known as the Empirical Phase Diagram (EPD), is a data visualization tool that is based on using data sets from multiple low-resolution biophysical techniques to construct a color coded diagram reflecting the different structural states that a protein would experience during at least two applied stress conditions (e.g., pH, temperature, ionic strength, protein concentration, etc.). Its application for formulation screening purposes for protein based drugs and vaccines has subsequently been extensively evaluated (Maddux et al., 2011). The initial idea of developing such a methodology stemmed from the fact that most comparative experiments using protein thermal melting curves conventionally use visual assessment and *T*_m_/*T*_onset_ values to determine differences between the different formulations under comparison. This makes data interpretation across multiple samples difficult and potentially highly subjective. Using bovine granulocyte colony stimulating factor (bGSCF) as a model protein combined with second derivative absorbance spectroscopy as a model biophysical method, the physical stability of the protein was characterized across multiple pH and temperature conditions, and the first EPD was constructed (Kueltzo et al., 2003). This EPD was able to detect six different structural (non-thermodynamic) phases that the protein experienced under a combination of the stress variables of temperature and pH. By visual observation of the individual data sets, these states were not easily detected.

An EPD analysis involves the collection of protein physical stability data under different experimental conditions using multiple low-resolution biophysical techniques. The stability data from the experimental conditions and techniques are then entered into *m* × *n* input matrix in which *m* represents the experimental techniques (i.e., circular dichroism, fluorescence spectroscopy, turbidity, etc.) and *n* represents the experimental conditions (i.e., number of pH values × number of temperature measurements). After data normalization, singular value decomposition (SVD) of the *m* × *n* experimental data matrix is calculated to extract three orthogonal basis vectors associated with the three largest singular values. These results are then mapped to red-green-blue (RGB) color scheme that can be visualized as a function of the stress conditions (e.g., temperature and pH). After an EPD is generated, what remains is the interpretation of the change of colors of the different areas of the EPD. The colors of the different areas within an EPD have no physical meaning. Instead, the change in color signifies a structural change in the protein as detected by the data from the individual experimental techniques. A scientist must refer to the original experimental biophysical data and consider the physical process that generated regions with different colors for further interpretation of the molecular origin of the color change.

**Figure 2:**
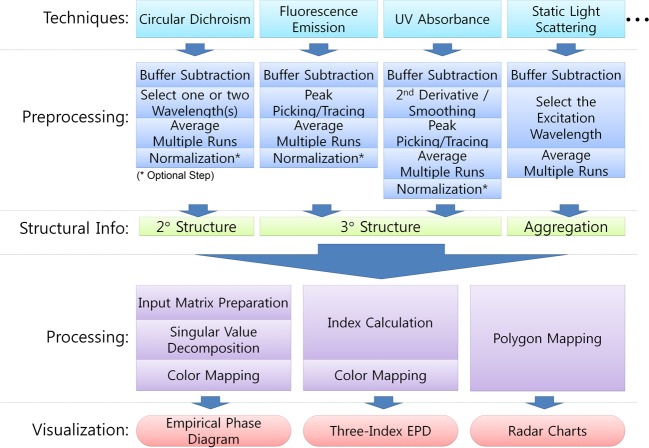
**Typical procedure for collection of biophysical stability data sets and construction of Empirical Phase Diagram (EPDs), Three-Index EPDs, and radar charts for data visualization and analysis**.

The EPD method employs SVD and linear algebra to extract distinctive patterns from a large collection of data. The EPD method requires a number of steps as described in Figure 2. First, the data must be extracted and stored from each instrument. Second, the data should be preprocessed to correctly represent macromolecular behavior. Care should be taken during pre-processing, since each physical method may require different types of pre-processing, and their results could directly impact the output observed in the EPD. This preprocessing may include buffer subtraction, smoothing, normalization, and peak picking. For example, peaks are picked and traced from Trp fluorescence under various environmental conditions. Since the maximum peak position of Trp fluorescence is closely related to the microenvironment around Trp residues, the peak position shift often indicates conformational changes in the macromolecule. Thus, the choice of peak picking algorithms, combined with the subsequent functions described next, can impact the resulting EPD and its interpretation of the macromolecular behavior. Third, preprocessed data should be organized into a matrix that will then be used as an input to SVD. The rows of the input matrix consist of samples under various environmental conditions. The columns are results from the experimental techniques. Finally, the three largest components from SVD results are selected and visualized as a two-dimensional colored diagram. The three largest components are the three left-singular vectors corresponding to the three largest singular values. Each vector is mapped to RGB colors; therefore, the combination of three produces a single color for each sample condition. The final EPD can be obtained by rearranging mapped colors along with two-dimensions of environmental variables.

EPDs have been extensively used in formulation development and stabilization studies with a wide variety of protein based drugs and vaccines as reviewed in detail elsewhere (Maddux et al., 2011). Since these EPD experiments have been carried out with a wide variety of proteins, and with many different type of biophysical techniques, the opportunity for improvements in the methodology have also been identified. For example, the use of color as a means to display patterns inherently possesses several disadvantages. The color itself in the EPD is automatically chosen; therefore, there is no explicit and common relationship between a color and the protein's structural state. This produces difficulties in interpretation of the EPD because the meaning of the color cannot be directly used to analyze experimental data. In addition, this method is difficult for people with vision color deficiencies. To overcome these disadvantages, several new data visualization approaches (Kim et al., 2012; Iyer et al., 2013) have been recently introduced.

First, instead of analyzing all experimental data together to construct an EPD, each method used to determine secondary, tertiary, and quaternary structural changes is analyzed separately and mapped to a fixed color set. In this manner, a three-index EPD can show structural changes in terms of defined colors. For example, the color yellow is always used to display the native state. The color blue is for the aggregated state and the color black for the completely unfolded state without any aggregation. The predefined colors enable intuitive analysis of not only a single, but also a collection of three-index EPDs at the same time (e.g., comparison of mutants). This three-index EPD method can be extended to visualize data from any three biophysical techniques, if a proper color legend is provided.

Second, Radar charts and Chernoff face diagrams have been utilized as color-free data visualization tools. They use certain icons such as polygon axes and facial expressions, respectively, to display large data sets under given conditions (Kim et al., 2012). They can accommodate a larger number of different data sets explicitly in a diagram compared to the original EPD method. The major idea of radar charts is to arrange multiple axes, each representing a different experimental technique, at evenly spaced angles to form a polygon. Radar charts are composed of multiple polygonal figures arranged in two dimensional coordinates of environmental stress conditions (e.g., temperature and pH). Each polygonal figure displays the experimental data from multiple instruments at the given stress conditions. Data from each instrument can be normalized to have values between zero and one. Then the value is mapped to a selected axis in the polar coordinate where zero is assigned to the center of the circle while one to the circumference. This mapping can be flipped (e.g., zero to circumference and one to center) if it is necessary to represent the native state of the protein as polygons with the smallest area. In this manner, the structurally altered state would be represented by polygons with larger area. Therefore, the increasing polygonal area indicates transitions from the native state to structurally altered states. Unlike EPDs, radar charts have the advantage of displaying protein physical stability results from a larger number of experimental techniques, allowing the evaluation of protein structural integrity and conformational stability over a wide range of methods and experimental conditions (Kim et al., 2012).

The Chernoff face diagrams utilize facial expressions instead of the polygons used with radar charts. Data sets from each instrument are also normalized to have values between zero and one. Then the value is mapped to a pre-defined facial feature such as the angle of eye-brows, the size of eye pupil, and the angle of mouth. These facial features are designed to express a variety of parameters with distinctive and easily recognizable faces. The data mapping can be adjusted to make the native state of the protein have smiley faces. The structurally altered state may have other facial expressions such as angry faces. Therefore, changes in facial expression indicate structural transition. Although the Chernoff face diagrams may not be appropriate to precisely visualize quantitative data as compared to radar charts or EPDs, they have strengths to make communication with non-scientific audiences more intuitive and easier (e.g., “In these conditions, the protein is happy.”).

Finally, comparative signature diagrams (CSDs) (Iyer et al., 2013) are designed to visualize statistically significant differences in biophysical stability data sets between two samples. The mean and standard deviation of multiple datasets from the same biophysical techniques for the two target samples to be compared are calculated first for each variable such as wavelength and temperature. The difference in means has the limiting normal distribution as the number of datasets approaches infinity. The threshold value such as three times the standard deviation of mean difference is typically given to decide statistically significant differences. Any mean difference within the threshold range will be discarded. Only statistically significant mean differences are plotted in the two dimensional space as contour lines and colors, whose two axes are the selected variables (e.g., wavelength, temperature, etc.). Data from multiple biophysical techniques can be overlaid in the same diagram by sharing the same temperature axis while each technique-specific axis such as wavelength is normalized accordingly.

## Case studies using data visualization techniques to compare protein physical stability profiles

The case studies presented in this section exemplify the utility of high throughput biophysical methods and data visualization tools to characterize protein physical stability. In these case studies, the structural integrity of several model proteins such as acidic fibroblast growth factor-1 (FGF-1), an IgG1 monoclonal antibody, and granulocyte colony stimulating factor (GCSF) were characterized using various biophysical methods as a function of temperature and solution pH. The large physical stability data sets obtained were combined into various types of diagrams for data visualization (Maddux et al., 2011; Kim et al., 2012; Iyer et al., 2013). Examples include EPDs, radar charts and CSDs as described above. The possible utility of these data visualization tools in future analytical comparability studies is considered.

### Case study: different mutants of acidic fibroblast growth factor (FGF-1)

Alsenaidy et al. (2012) examined the utility of EPD based approaches as an analytical tool to compare the conformational stability profiles of various acidic fibroblast growth factor-1 (FGF-1) mutants (Alsenaidy et al., 2012). The low intrinsic stability of FGF-1 has been identified as one of the key challenges in the development of FGF-1 as a successful pro-angiogenic drug candidate for the treatment of ischemic diseases and wound healing. Polyanion like heparin produce a substantial increase in the physico-chemical stability and activity of FGF-1 by binding to the native form of the protein (Mach et al., 1993; Tsai et al., 1993). The addition of complex molecules like heparin, however, is associated with additional complications such as enhanced cost, immunogenicity and intrinsic pharmacological activity. As an alternative, a mutation based approach directed toward enhancing the intrinsic stability of FGF-1 without adding exogenous excipients was evaluated to reduce the dependency of FGF-1 on heparin or other polyanions for stability and activity (Alsenaidy et al., 2012). A series of FGF-1 mutants were developed by mutating human FGF-1 to enhance its intrinsic stability and obtain candidates with a stability profile similar to wild type (WT) FGF-1 in the presence of heparin. The thermodynamic stability and biological evaluation parameters reported in the literature for WT and multiple FGF-1 mutants are summarized in Table 1. The EPD evaluation for K12V/C117V/P134V (mutant H in Table 1) and its comparison to WT-FGF-1 in the presence and absence of heparin will be described in detail as an example. This mutant has shown a significant increase in the thermostability (−ΔΔG = 19.1 kJ/mol) and activity (EC50 = 1.80 ± 0.90 ng/mL) compared to WT FGF-1 without heparin (Sample A in Table 1).

**Table 1:** **Summary of conformational stability (ΔΔG) and biological activity (EC50, half-life, heparin binding) values for wildtype (WT) FGF-1 and its mutants**.

**FGF-1 protein (reference)**	**Symbol**	**ΔΔG (kJ/mol)**	**EC50 (ng/mL)(−) heparin**	**EC50 (ng/mL) (+) heparin**	**Half-life (*h*)**	**Heparin binding**
**Group I**						
WT (without and with heparin)	A and B	-	58.4±25.4	0.48±0.08	1.0	Yes
L26D/H93G (Lee et al., 2008)	C	−0.9	N.A.	N.A.	N.A.	Yes
C83T/C117V/K12V (Lee and Blaber, 2009a)	D	−1.9	0.93±0.25	0.36±0.12	40.4	Yes
P134V/C117V (Dubey et al., 2007)	E	−8.8	46.8±6.7	N.A.	N.A.	Yes
K12V/C117V (Dubey et al., 2007)	F	−9.3	4.2±1.7	N.A.	N.A.	Yes
A66C (oxi) (Lee and Blaber, 2009b, 2010)	G	−10.2	5.43±3.96	0.36±0.20	14.2	Yes
K12V/C117V/P134V (Dubey et al., 2007)	H	−19.1	1.80±0.90	N.A.	N.A.	Yes
**GROUP II**						
C83T/C117V/L44F/F132W (Lee and Blaber, 2009a)	I	−0.4	0.74±0.19	0.51±0.15	42.6	Yes
SYM6ΔΔ/K12V/P134V (Lee et al., 2011)	J	−35.0	741±302	N.A.	N.A.	No
Symfoil-4P (Lee and Blaber, 2011)	K	−44.1	N.A.	N.A.	N.A.	No
SYM10ΔΔ (Lee et al., 2011)	L	−47.0	N.D.	N.A.	N.A.	No

*See indicated references for values of individual mutants. Reproduced from Alsenaidy et al. (2012) with permission from John Wiley and Sons*.

Both WT and mutant FGF-1 proteins were characterized as a function of solution pH (3–8) and temperature (10–90°C) using various biophysical techniques. Far-ultraviolet circular dichroism (far-UV CD) was used to characterize secondary (and tertiary) structural changes in the WT and mutant FGF-1 at each pH value. The CD signal at 228 nm, presumably reflecting a signal from a combination of β-turns, loops and aromatic side chains, was monitored as a function of temperature from 10 to 90°C. Intrinsic fluorescence measurements were carried out to monitor tertiary structural changes. FGF-1 contains eight Tyr and one Trp and the latter is quenched in the native state. Samples were excited at 280 nm and the ratio of fluorescence intensity at 305 and 330 nm (I_305_/I_330_) was monitored as a function of temperature to reflect the fluorescence signals from both Trp and Try residues. The intensity at 305 nm was primarily due to Try residues whereas 330 nm corresponds to Trp residues. Because a single Trp residue is quenched in the native conformation of FGF-1, higher values of I_305_/I_330_ indicate native like structure whereas low values reflect an altered conformation. Extrinsic fluorescence measurements using 1-Anilino-8-naphthalnesulfonate (ANS) were also performed to monitor the exposure of apolar sites as an indicator of conformational alteration in the protein. ANS fluorescence intensity was measured at 480 nm as a function of temperature. Aggregation behavior of the protein at different pH values was evaluated by measuring static light scattering at 280 nm as a function of temperature.

**Figure 3:**
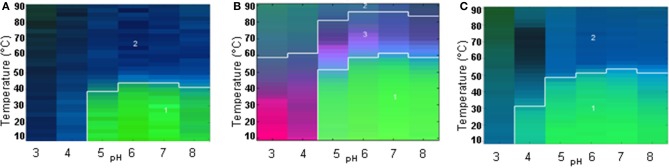
**Empirical Phase Diagrams (EPDs) for comparative analysis of conformational stability of FGF-1 and its mutant**. The EPDs were developed for wildtype FGF-1 **(A)**, wildtype FGF-1 with heparin **(B)** and K12V/C117V/P134V mutant of FGF-1 (no heparin) **(C)**. Stability data as function of pH and temperature were collected from the following methods: intrinsic fluorescence intensity ratio at two wavelengths (I305/I330 nm), CD at 228 nm, static light scattering (SLS) and ANS fluorescence intensity. Reproduced from Alsenaidy et al. (2012) with permission from John Wiley and Sons.

The large data sets obtained from all the techniques for each pH value from 3 to 8 were summarized into an EPD. Two distinct regions were observed in the EPD for WT-FGF-1 without heparin (Figure 3A). The green area (region 1) comprises neutral pH (~4.5–8) and low temperature (~10–40°C) and presumably reflects a more stable, native-like state of the protein. The blue area (region 2) reflects a conformationally altered and/or aggregated state. A structure transition was observed in the region between pH 6 and 7 at ~44°C. A third region manifested as a light blue region between the boundaries of region 1 and 2 was also present but wasn't well resolved. This region suggests the presence of a molten globule (MG)-like state (near native secondary structure, altered tertiary structure). As shown in Figure 3B, in the presence of heparin, an apparent boundary for region 1 (green area) shifts toward a higher temperature region. The area covered by region 2 (blue area) decreased and a third region (pink area), reflecting the presence of an MG-like state become more prominent. The structural transitions from native to altered conformations are observed at ~60°C which is ~16°C higher than the WT FGF-1 in the absence of heparin. A comparison between two EPDs for FGF-1 without (Figure 3A) and with heparin (Figure 3B) showed differences in various states of the protein. The native like state as reflected by region 1 (green area) is more prominent in the FGF-1 with heparin (Figure 3B). The altered conformation/aggregated state is also less evident and a higher structural transition temperature is observed for FGF-1 with heparin. This comparison suggests a better stability of FGF-1 in the presence of heparin than without heparin and is in agreement with the parameters shown in Table 1.

The EPD for K12V/C117V/P134V (mutant H in Table 1) again showed two regions, region 1 (green area) and region 2 (blue area) reflecting native-like and an altered/aggregated state of the protein, respectively (see Figure 3C). The area encompassing the native-like state (region 1) is more expanded than in the EPD for WT FGF-1 without heparin (Figure 3A). This result indicates the better stability of the mutant in the absence of heparin. The structural transition temperature is also observed at 52°C which is ~8°C higher than the WT FGF-1 without heparin. Taken together with the parameters in Table 1, comparative EPD analysis confirms that the mutant protein is in a stable and bioactive conformation that does not rely on presence of heparin. This study provides evidence that the EPD methodology can be a useful analytical tool in comparing the global physical stability profile of different FGF-1 mutants.

### Case study: different IgG1 mAb glycoforms due to deglycosylation

This second case study illustrates the applicability of a combination of data visualization techniques including EPDs and radar plots to the evaluation of the conformational stability profiles of different glycoforms of an IgG1 monoclonal antibody. The glycosylation pattern of monoclonal antibodies can play an important role their functionality and clearance from the body (Arnold et al., 2007), and can influence conformational stability and solubility (?Krapp et al., 2003; Wacker et al., 2011). In this study, Alsenaidy et al. (2013) evaluated changes in the structural and conformational integrity of various glycoforms of an IgG1 mAb generated by enzymatic deglycosylation to obtain partially and fully deglycosylated forms (Alsenaidy et al., 2013). A two-step method was adopted in this work to monitor the structural and conformational changes in the mAb glycoforms as function of solution pH and temperature. In the first step, the EPDs were generated over a wide range of solution conditions with a variety of analytical techniques. In the second step, a narrower temperature and pH range was selected along with employment of the most sensitive analytical techniques. A pH range of 4.0–6.0 with 0.5 increments was selected based on the initial, step-one experiments with completely glycosylated and deglycosylated mAbs. The techniques used to monitor changes in different structural and conformational features included far-UV CD for secondary structure, intrinsic tryptophan and extrinsic ANS/Sypro Orange fluorescence spectroscopy for tertiary structure, static light scattering for aggregation and DSC for overall structural stability. The physical stability data sets obtained from the different techniques were summarized into EPDs and radar charts for analysis and comparison of conformational stability profiles.

**Figure 4:**
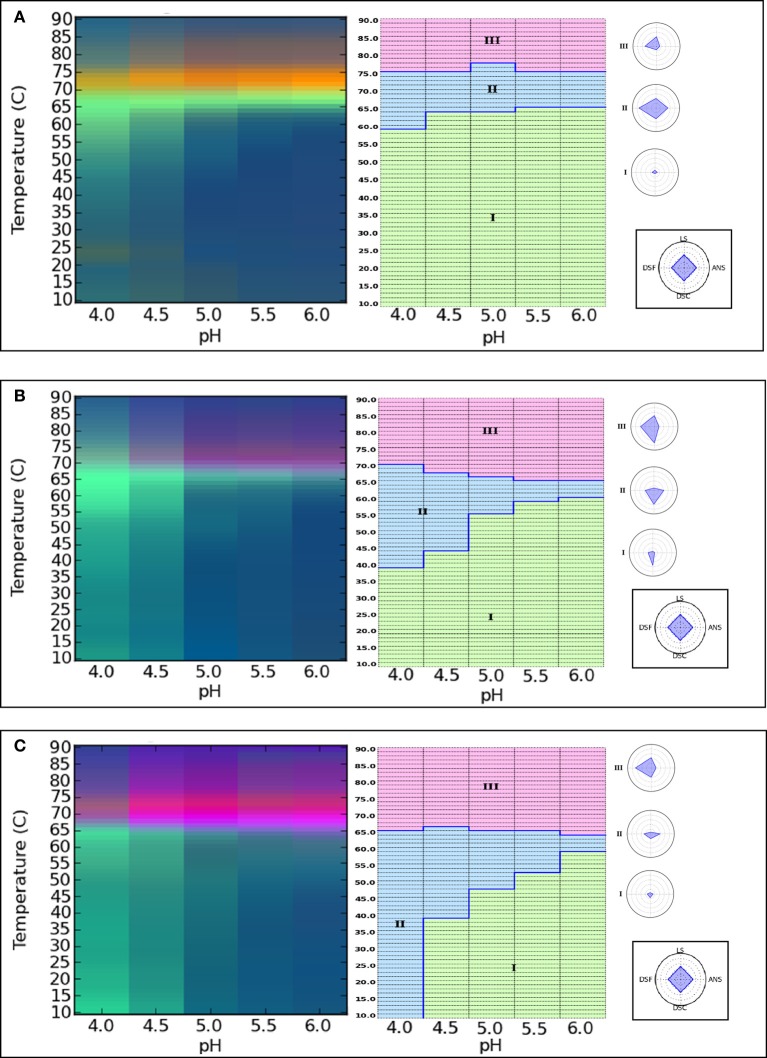
**Analysis of the conformational stability of IgG1 mAb glycoforms using Empirical phase diagrams (left panel) and Radar charts (right panel)**. The diagrams show conformation stability of fully glycosylated (control) **(A)**, partially deglycosylated **(B)** and fully deglycosylated **(C)** IgG1 mAb. Stability data as function of pH and temperature were collected from the following methods: differential scanning calorimetry, differential scanning fluorimetry, ANS fluorescence intensity and static light scattering. Reproduced from Alsenaidy et al. (2013) with permission from John Wiley and Sons.

As shown in Figure 4 (left panel), EPDs for the untreated, native mAb (control) (Figure 4A), partially deglycosylated (Figure 4B) and completely deglycosylated (Figure 4C) mAb were generated. The blue and green colors in the EPDs reflect regions where the IgG1 mAb is in a native-like and structurally alerted state, respectively. A third region of varying colors and intensity signifies aggregation or precipitation of the IgG1 glycoforms. The conformational stability can be compared among different IgG1 glycoforms using EPDs by comparing the location of the boundaries and area encompassed by the different color regions. Comparing blue regions which reflect the appearance of the native state of the protein across different EPDs, at pH 5.5 and 6, the structural transition temperature was 64°C in the case of untreated mAb (Figure 4A) and partially deglycosylated IgG1 (Figure 4B) whereas a value of 59°C was obtained for the completely deglycosylated form (Figure 4C). At pH 5.0, transition temperatures changed to 63 and 61°C for native and partially deglycosylated forms, respectively. At this pH, the EPD for the completely deglycosylated IgG1 showed additional color regions suggesting the presence of multiple conformationally altered states of the protein. The structural transition temperatures were further lowered to 50 and 55°C for untreated IgG1 at pH 4.0 and 4.5, respectively. At these pH values, the partially deglycosylated (Figure 4B) and fully deglycosylated (Figure 4C) forms showed additional colored regions reflecting different conformationally altered sates of these glycoforms. The presence of additional green region in fully deglycosylated mAb (Figure 4C) at pH 4 suggests more extensive structural/ conformational alterations even at lower temperatures.

Radar plots were also constructed (Figure 4, right panel) using the same biophysical stability data set used for the construction of the color EPDs. The structural transitions were obtained by using a k-Means clustering algorithm to identify the apparent phase boundaries as described in the previous section. Under the similar pH and temperature range used for the EPDs, similar regions of conformation and structure were identified in the radar plots. The region of native-like conformation/structure was 67, 52, and 40% for untreated, partially deglycosylated, and fully deglycosylated forms of the mAb, respectively. These results demonstrate that the physical stability profile (i.e., EPD and radar chart diagrams of pH vs. temperature) of this IgG1 mAb changed as a function of varying its glycosylation pattern in a pH dependent manner.

### Case study: different formulations of granulocyte colony stimulating factor (GCSF)

The third case study describes an alternative data visualization tool that involves a more rigorous mathematical treatment of the biophysical data to carry out a statistical comparison for comparability studies (Iyer et al., 2013). The CSDs were proposed as a way to measure the statistical significant difference among different sets of spectral data generated from various biophysical instruments used for protein structure/conformation characterization. This study illustrates construction of CSDs for the protein Granulocyte Colony Stimulating Factor (GCSF) in 16 different formulations. The protein formulations were characterized using far-UV CD, Trp intrinsic fluorescence, ANS extrinsic fluorescence and static light scattering, and the data as a function of pH and temperature were obtained for the construction of CSDs.

**Figure 5:**
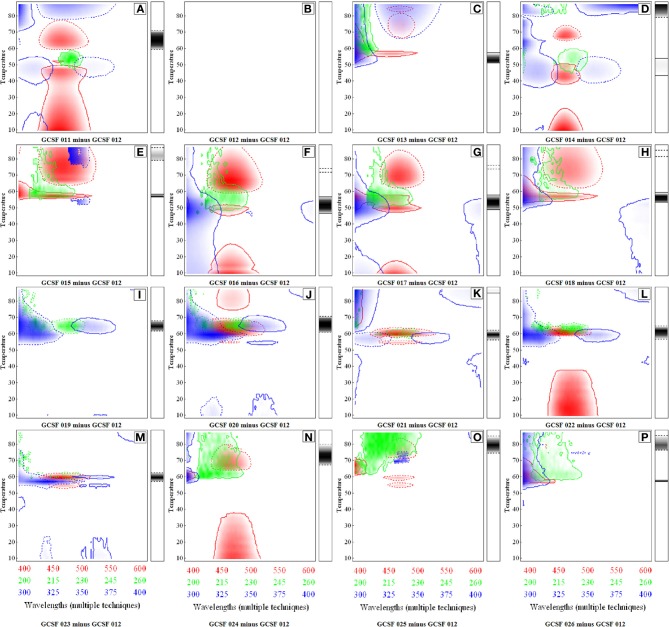
**Comparative Signature Diagrams (CSDs) for different formulations of Granulocyte Colony Stimulating Factor (GCSF)**. Formulation GCSF 12 was used as the control condition. Each diagram **(A–P)** represents the comparison of a particular formulation with GCSF 12. Red, blue, and green colors represent differences in spectra of ANS fluorescence, intrinsic fluorescence and CD, respectively. The side bars represent differences in aggregation pattern. The solid outline indicates positive differences and dashed outline negative differences. Reproduced from Iyer et al. (2013) with permission from John Wiley and Sons.

These CSD diagrams incorporate the entire spectrum and any significant differences between data sets are represented as colored regions. The x and y axis in CSDs represent different arbitrary values to signify the data from different techniques and formulation variables. As shown in Figure 5, CSDs were constructed for 16 different GCSF formulations listed in the Table 2. These diagrams were constructed at the 75th quartile using a difference of five standard deviations to show significant differences which are illustrated by various colors. Differences in CD spectra (200–260 nm) are represented by green color, intrinsic Trp fluorescence (300–400 nm) by blue color, and extrinsic ANS fluorescence (400–600 nm) by red color. The side bar reflects the differences seen in the aggregation behavior. The solid outline represents positive differences whereas dashed outline shows negative ones.

**Table 2:** **Composition of different formulations containing the protein granulocyte colony stimulating factor (GCSF)**.

**Formulation**	**Buffer**	**Buffer concentration (mM)**	**pH**	**Excipient**	**Excipient concentration (%)**
GCSF011	No	No	4.5	Tween-80	0.05
GCSF012	No	No	4.5	Tween-80	0.005
GCSF013	No	No	5	Tween-80	0.005
GCSF014	No	No	5	Tween-80	0.05
GCSF015	Citrate	20	4.5	Tween-80	0.005
GCSF016	Citrate	50	4.5	Tween-80	0.05
GCSF017	Citrate	20	5	Tween-80	0.05
GCSF018	Citrate	50	5	Tween-80	0.005
GCSF019	No	No	4	HPBCD	5
GCSF020	No	No	4	HPBCD	1
GCSF021	No	No	4.5	HPBCD	1
GCSF022	No	No	4.5	HPBCD	5
GCSF023	Acetate	20	4	HPBCD	1
GCSF024	Acetate	100	4	HPBCD	5
GCSF025	Acetate	20	4.5	HPBCD	5
GCSF026	Acetate	100	4.5	HPBCD	1

*The physical stability of GCSF in the different formulations was measured by multiple biophysical methods and results compared using comparative signature diagrams (CSDs) as shown in Figure 5 Reproduced from Iyer et al. (2013) with permission from John Wiley and Sons*.

*HPBCD, Hydroxypropyl Beta Cyclodextrin*.

As shown in Figure 5, a comparative analysis of the different GCSF formulations compared to a control formulation (GCSF 012 in Table 2) is presented. For the current discussion, only Figure 5A (formulation GCSF 011 minus GCSF 012) is described in detail. The differences in CD spectra (secondary structure) as depicted by green color were seen at ~215–230 nm where GCSF 12 shows relatively larger changes in secondary structure above 60°C. The tertiary structural changes as illustrated by the blue color were seen in different temperatures regions. In 300–325 and 350–375 nm regions of the spectra at ~40–45°C, light blue colored regions reflect minor conformational differences of the protein in the two formulations. The strong blue color in the ~300–310 nm range at above 70°C reflects the higher aggregation propensity of the protein in the GCSF 011 formulation. This effect is supported by a strong negative difference in the light scattering data between the two formulations as represented by the side bar with dotted outline in Figure 5. Finally, the red color in the lower temperature range shows the differences in ANS fluorescence in the two protein formulations. This fluorescence difference was suspected to be due to ANS bound to polysorbate-80 which was present at a high concentration (0.05%) in GCSF 011. The red color in higher temperature regions reflects the enhanced binding of ANS to the protein in control formulation. As a control, Figure 5B depicts a CSD diagram comparing the same formulation to itself. The absence of colored regions shows the internal consistency of method.

Looking across the various formulations in Figure 5, Formulation GCSF 019 (Figure 5I) and GCSF 020 (Figure 5J) showed the least differences whereas GCSF 24 (Figure 5N), GCSF 25 (Figure 5O) and GCSF 26 (Figure 5P) reflects the largest differences in structures/conformation based on the CSD analysis. This technique shows its potential to detect significant differences in protein spectra among different formulations under varying conditions of pH and temperature. Further work using different analytical techniques and additional proteins, however, is required to better establish the use of CSDs in analytical comparability studies.

### Case study: different aggregation profiles for an IgG1 mAb

The comparisons of impurity profiles between samples can be another important aspect of comparability assessments. One key product related impurity is the formation of aggregates and particles in protein formulations during storage (Wang, 2005; Hamada et al., 2009; Wuchner et al., 2010). A careful analysis of particle/aggregate profiles can be an important component of comparability studies where changing the pharmaceutical dosage form during development can lead to changes in the formation of these unwanted species (Lubiniecki et al., 2011; Federici et al., 2013). Evaluation of the effects of various stress and solution conditions and excipients on particle/aggregate formation, however, remains challenging due to lack of suitable analytical tools for comprehensive analysis of a large amount of data generated by various methods used in particle size characterization (Wang et al., 2013b).

In this case study, Kalonia et al. (2013) have evaluated radar charts as a novel data visualization tool to compare aggregate and particle formation profiles for an IgG1 monoclonal antibody in different formulations upon subjecting to different stress conditions. Effects of various solution parameters such as pH and NaCl concentration on size, concentration, kinetics and morphology of subvisible IgG mAb particle formation after shaking, stirring and heating were analyzed by Micro Flow Imaging (MFI). MFI is a digital imaging technology that generates tens to hundreds of thousands of images of protein particles in solution. These large data sets can be difficult to analyze, and in this work, data generated by MFI was incorporated into radar chart arrays to better assess and compare the size, number and morphology of protein particles formed in different formulations exposed to different stress conditions.

**Figure 6:**
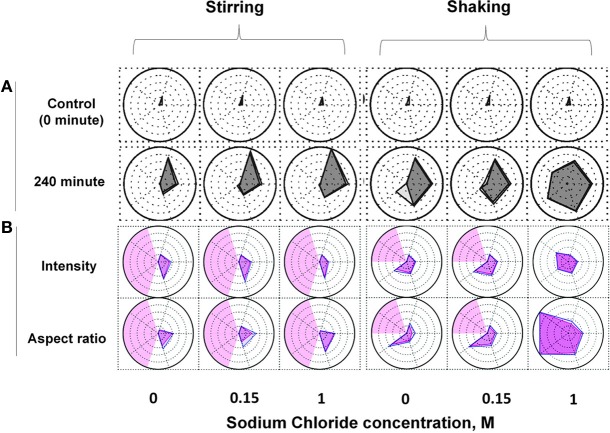
**Radar chart analysis of subvisible particles in IgG1 mAb solution after stirring and shaking stresses in the presence of varying amount of sodium chloride**. The size and number **(A)** and morphology **(B)** of subvisible particles were measured by Micro Flow Imaging (MFI). Each axis in an individual radar chart represents particles of various size ranges (clockwise from top: 2–5, 5–10, 10–25, 25–50 and 50–70 μm). Each ring positioned from the center to periphery displays in **(A)** 10 fold increase in particle concentration from <10 (center) to >10^6^ (edge) particles/ml, and in **(B)** 0.1 increments in morphological parameters (aspect ratio or intensity/1000) from <0.35 (center) to >0.85 (edge). The outer polygons show mean values whereas inner polygons represent mean minus 1SD (standard deviation) in **(A)** and mean plus 1SD in **(B)**. The light red shaded regions in **(B)** represent areas with insufficient number of particles to report results (less than 25). Reproduced from Kalonia et al. (2013) with permission from John Wiley and Sons.

As shown in Figure 6, a series of radar charts were generated to compare the effect of two different stress conditions and a varying amount of NaCl on the size and number of IgG1 subvisible particles formed (Figure 6A) and the morphology of these particles (Figure 6B). Unstressed mAb samples show approximately 100–3000 subvisible particle across the different salt concentrations (Figure 6A, upper panel). Shaking and stirring of samples for 240 min resulted in the formation of a large amount of particles of varying number and size (Figure 6A, lower panel). Particles of larger size (25–50 and 50–70 μm) were formed in the samples subjected to shaking stress compared to stirred samples (particle size primarily in the 2–25 μm range). The formation of particles/aggregates was significantly influenced by the concentration of salt. For example, after stirring for 240 min, samples without salt showed less than 50,000 particles/ml whereas 300,000 and >10^6^ particles/ml of 2–5 μm size range were observed in the presence of 0.15 M and 1 M NaCl, respectively. One important feature of the radar charts is that they include the statistical variability in the data as shown in the shaded regions in Figure 6A.

Radar charts were also constructed for morphology analysis of subvisible IgG1 particles formed under the above mentioned solution and stress conditions. As shown in Figure 6B, the average particle aspect ratio (a measure of particle elongation) and intensity (a measure particle transparency) remained constant upon stirring for 240 min across the different salt concentrations. In the case of shaking for 240 min, the average aspect ratio of particles decreased in the presence of 1 M salt. An average aspect ratio of less than 0.35 was calculated for subvisible particles larger than 25 μm indicating the formation of fiber-like particles under these conditions. Compared to no salt or 0.15 M salt samples, an increase in intensity of the particles in the size range of 25–50 μm was observed in mAb samples shaken in the presence of 1 M salt (Figure 6B).

Radar charts were also prepared for analysis of data from multiple particle sizing/counting instruments which cover a wider range of mAb particle sizes. Size exclusion chromatography (SEC) was used to measure soluble aggregates, resonance mass measurements (RMM) to measure sub-micron particles (~0.3–2 μm) and MFI to measure sub-visible particles (2–70 μm). These “multiple-instrument radar charts” were prepared for the heat and stirring induced aggregation of the mAb under varying salt concentrations and the mechanistic basis and kinetics of aggregates/particles formation was evaluated. Such types of data presentation further diversify the applicability of the radar chart based data visualization technique in comprehensive characterization of different sizes of aggregates/particles in various formulations under a variety of stress conditions.

This case study illustrates the capability of using radar charts to summarize and compare large data sets from MFI analysis of particle size distribution, concentration, and morphology across different protein formulations in a single figure. This work also demonstrates the potential utility of radar chart analysis to compare the profiles of aggregate and particle formation of varying size ranges and morphologies as measured by a variety of analytical methods, across different protein samples in analytical comparability assessments.

## Summary

The analysis of protein higher-order structure is an essential part of analytical comparability assessments of biopharmaceutical drug candidates. Although there are a number of higher resolution analytical tools potentially available to examine and compare the higher-order structure of protein molecules, many of these techniques have practical limitations in their routine use including technical complexities, low-throughput, high costs and difficult interpretability. Additionally, the size and concentration of a protein as well as the presence of various additives in the formulation (which can interfere with some of these assays) further restrict their use. While lower resolution biophysical techniques are more readily available to analyze various structural aspects of protein molecules in pharmaceutical formulations, such methods may be unable to detect minor structural alterations in protein molecules even if they have significant implications for their functionality. In this review, we have illustrated that the biophysical characterization of various protein molecules (subjected to different environmental stresses, e.g., solution conditions, pH, temperature, agitation), by using a combination of lower resolution, high-throughput techniques combined with various data visualization tools, can detect physical stability differences in proteins with minor structural/conformational changes. The case studies of FGF-1 mutants and differentially glycosylated IgG-1 mAbs demonstrated the application of this approach to compare the effect of relatively minor modifications in protein structure on their conformational stability. The GCSF case study illustrated a data visualization technique to statistically compare the structural/conformational stability differences measured with the same protein in different formulations. The final case study presented a radar chart data visualization technique to more comprehensively characterize protein aggregate and particle formation as generated by two different agitation stresses applied to IgG1 mAb solutions containing varying levels of sodium chloride. In conclusion, characterization of the physical stability of proteins using various lower resolution, higher throughput biophysical techniques, combined with different environmental stresses and data visualization tools, can potentially as serve as a useful tool to evaluate the higher-order structural integrity of proteins for comparability purposes.

### Conflict of interest statement

The authors declare that the research was conducted in the absence of any commercial or financial relationships that could be construed as a potential conflict of interest.
